# Epidermal Growth Factor Receptor Emerges as a Viable Target for Reducing Tumorigenicity of MDCK Cells

**DOI:** 10.3390/genes15091208

**Published:** 2024-09-14

**Authors:** Di Yang, Yuejiao Liao, Lingwei Huang, Jiachen Shi, Jiamin Wang, Zilin Qiao, Zhongren Ma, Sijiu Yu

**Affiliations:** 1 College of Veterinary Medicine, Gansu Agricultural University, Lanzhou 730070, China; xbmzyd@163.com (D.Y.); lyj1997052@163.com (Y.L.); 2 Engineering Research Center of Key Technology and Industrialization of Cell-Based Vaccine, Biomedical Research Center, Northwest Minzu University, Lanzhou 730030, China; vivhuanglw@163.com (L.H.); shijiachen2022@163.com (J.S.); jiaminwang1987@163.com (J.W.); qiaozilin@xbmu.edu.cn (Z.Q.); mzr@xbmu.edu.cn (Z.M.); 3 Gansu Tech Innovation Center of Animal Cell, Biomedical Research Center, Northwest Minzu University, Lanzhou 730030, China; 4 Department of Experiment and Teaching, Northwest Minzu University, Lanzhou 730030, China; 5 Key Laboratory of Biotechnology and Bioengineering of State Ethnic Affairs Commission, Biomedical Research Center, Northwest Minzu University, Lanzhou 730030, China

**Keywords:** MDCK cell line, epidermal growth factor receptor (EGFR), tumorigenicity, influenza vaccine, cell substrates

## Abstract

The MDCK cell line is perceived as better than the embryos of hen eggs for the production of influenza vaccines, but the tumorigenicity of these cells is concerning. Epidermal growth factor receptor (EGFR) is likely to be a crucial target that contributes to the tumorigenicity of MDCK cells. In this study, EGFR-knockdown and EGFR-overexpression cell lines were established. EGFR’s influence on cell growth, migration, clonogenic ability, and flu virus susceptibility was evaluated in vitro, and its role in cell tumorigenicity was examined in nude mice. GST pull-down coupled with mass spectrometry (MS) and bioinformatics analysis identified EGFR-interacting proteins. The expression levels of these proteins, as well as those of PI3K–AKT- and MAPK–ERK-signaling-pathway-related molecules, were confirmed at both gene and protein levels. The result indicates that EGFR overexpression can enhance cell proliferation, migration, and clonal formation; EGFR knockdown could effectively curtail tumorigenesis and amplify the titers of influenza viruses in MDCK cells. An analysis of the underlying mechanism identified a total of 21 interacting proteins implicated in tumor formation, and among these, AKT1, CDK4, GNB2, and MAPK8 were confirmed at both gene and protein levels. EGFR can activate key factors of the PI3K–AKT signaling pathway, AKT and PI3K, and promote their phosphorylation levels. Consequently, we concluded that EGFR interacts with GNB2, facilitating transmembrane signal transduction, activating the PI3K–AKT signaling cascade, controlling cell cycle alterations, stimulating cell proliferation, and promoting tumorigenesis.

## 1. Introduction

Influenza is a significant global public health concern, as seasonal influenza virus infection results in approximately 3 to 5 million severe cases and 290,000 to 650,000 fatalities each year [1,2]. Vaccination serves as one of the most effective medical strategies for the prevention of influenza virus infection. Over the past two decades, substantial advancements have been made in the development of vaccines. Cell-based vaccines are developed by using Madin–Darby canine kidney (MDCK) cells for the propagation of candidate vaccine viruses (CVVs) instead of chicken eggs, in order to overcome the potential for mismatch due to egg-adaptive mutations [3]. The Madin–Darby canine kidney (MDCK) cell line was isolated from the normal kidney of a female cocker spaniel by renowned scientists S.H. Madin and N.B. Darby. This cell line exhibits remarkable characteristics that allow the continuous passaging and robust replication of influenza viruses of various subtypes, and is extensively utilized as a cell substrate for the production of influenza vaccines and various other biological products [4].

Cells are employed for the production of biological products. Many continuous cell lines (CCLs) (e.g., MDCK, BHK-21, CHO, and HeLa) are characterized as tumorigenic due to their inherent ability to generate tumors in immunocompromised animals such as rodents [5,6]; Vero cells are tumorigenic at high generations and can be used in vaccine production, but their use for vaccine generation is limited [7,8]; and the pluripotent nature of stem cells gives them the ability to form tumors in immunodeficient mice, even though they exhibit a diploid karyotype [5]. Cell lines that have undergone multiple passages have lost the characteristic of contact inhibition and acquired the capacity for indefinite in vitro proliferation, thereby posing a risk of malignant transformation and tumorigenicity. Various national regulatory agencies, such as the European Medicines Agency (EMA), the U.S. Food and Drug Administration (FDA), and China’s National Medical Products Administration (NMPA), have established technical requirements for characterizing and testing cell banks employed in production. Therefore, the detection of tumorigenicity in CCLs and the reduction or elimination of tumorigenic cell factors during the manufacturing process can facilitate product approval. MDCK cells display an infinite capacity for self-renewal, and genetic modifications may occur during passage leading to the tumorigenic phenotype of the cell substrate [9]; the tumorigenic property of MDCK cells may be attributed to cell heterogeneity and epithelial–mesenchymal transition (EMT) [10]. If steps such as inactivation and purification are not performed, the risk of using MDCK cells to produce live attenuated vaccines is higher because of their tumorigenicity [11]. On the other hand, the utilization of tumorigenic cell lines may allow the transmission of potential carcinogenic factors such as oncogenic viruses or gene sequences that may transform host cells [9,12]. Therefore, it is necessary to explore the mechanism of cell tumorigenesis.

The emergence of high-throughput sequencing technology has opened up new avenues for scrutinizing molecular-level signals associated with tumor formation, thereby facilitating a more precise evaluation of the tumorigenic potential of cell lines. In our previous studies, we derived non-tumorigenic MDCK cell clones through cell cloning, which is obviously required for vaccine production [13]. We conducted a comprehensive transcriptome analysis of the parental MDCK cells from ATCC and non-tumorigenic clonal cells, identifying several pivotal genes implicated in tumorigenesis [13,14,15]. One of these genes was epidermal growth factor receptor (EGFR), a member of the ErbB family of receptor tyrosine kinases (RTKs) [13]. Furthermore, our analysis of differentially expressed long noncoding RNAs (lncRNAs) revealed the intriguing finding that the lncRNA MSTRG.1056.2 had the ability to directly regulate the expression of ERBB3, another member of the ErbB family of RTKs [14]. These discoveries suggested that the ErbB family of RTKs might contribute to the development of cellular tumors in the MDCK cell line. Consequently, EGFR would emerge as a critical target gene that significantly contributes to the tumorigenicity of MDCK cells. Future studies aiming to elucidate the role of gene regulation in cellular tumorigenesis and its underlying mechanism may be facilitated by the targeted modification of this gene. In this study, we aimed to determine the effect of EGFR on the tumorigenicity of MDCK cells and investigate strategies to overcome this problem.

## 2. Materials and Methods

### 2.1. Cells, EGFR shRNA, and EGFR Overexpression Plasmid

One strain of MDCK cells (ATCC CCL-34) was acquired from American Type Culture Collection (ATCC), and the main bank cells were named as MDCK-M-60. The MDCK working bank cells (MDCK-WBC) used in the experiment were established by the main bank cells. Following the protocol established by Ma et al. [13], monoclonal cell lines MDCK-CA005, MDCK-CB057, and MDCK-CL09 were derived from the MDCK working cell bank. Upon validation, it was observed that MDCK-WBC formed nodules at the injection site in 4~6-week-old nude mice (10/10, 100% tumorigenicity), whereas the cloned cell lines MDCK-CA005, MDCK-CB057, and MDCK-CL09 did not demonstrate tumorigenic potential (0/10, 0% tumorigenicity) [13]. All aforementioned cells were propagated in DMEM supplemented with 10% fetal bovine serum (FBS, Cellmax, Beijing, China).

Based on the *EGFR* gene sequence (Gene ID: 404306), the siRNA target sequence 5′-GGAGATAAGCGATGGAGATGT-3′ was designed, and the recombinant short hairpin RNA (sh-RNA) plasmid was generated by fusion with the linearized GV298 vector (the map for plasmid GV298 is shown in Figure S1A). *EGFR* gene sequence design and synthesis were based on the following: EGFR-P1, 5′-AGGTCGACTCTAGAGGATCCCGCCACCATGCGCCCCCCGGGGCCCGTGGGAGCC-3′; and EGFR-P2, 5′-TCCTTGTAGTCCATACCGGTCGCTCCAATAAACTCACTGCTTGGTGGCGCTACCC-3′. The target fragment was synthesized by PCR amplification, and the recombinant EGFR overexpression plasmid was generated by fusion with the linearized GV492 vector (the map for plasmid GV492 is shown in Figure S1B). The parameters for the PCR were as follows: 98 °C for 5 min (one cycle), 98 °C for 10 s, 55 °C for 10 s, and 72 °C for 98 s (30 cycles in total), 72 °C for 8 min (one cycle), and 4 °C (one cycle).

Recombinant shRNA-EGFR lentivirus and EGFR-overexpressing lentivirus were constructed using the lentivirus packaging plasmid by GeneChem Co., Ltd., Shanghai, China. The negative-control lentiviruses (lv-GFP) and RNAi negative-control (5′-TTCTCCGAACGTGTCACGT-3′) lentiviruses were provided by GeneChem.

### 2.2. Lentivirus-Transfected Cells

MDCK cells were cultivated in six-well plates at a density of 1 × 10^5^ per well and exposed to recombinant lentivirus at a multiplicity of infection (MOI) of 100 after 16 h of incubation at 37 °C. Virus-infected cells were harvested 48 h later and inoculated into T25 cell vials. Positive cells were screened using DMEM supplemented with 10% FBS and 0.4 µg/mL puromycin at 37 °C in an atmosphere with 5% CO_2_. Once the fusion degree reached approximately 70~80%, the cells were serially diluted 1:6 and screened for 3~4 generations. The screened cells were enzymatically digested, counted, and diluted to 1 × 10^3^ cells/mL using complete medium, 100 µL of the cell suspension was transferred to 20 mL of complete medium containing 0.4 µg/mL puromycin, and the resulting cell suspension was further diluted to 5 cells/mL. A U-shaped 96-well plate was utilized for the inoculation of 200 µL of the cell suspension per well, and the inoculated cell density was 1 cell/well. The inoculated cells were cultivated in a 5% CO_2_ incubator at 37 °C for 4 h. The single-cell wells were individually marked under the microscope, and the growth of single cells was monitored daily. The solution was refreshed on the 4th and 8th days, and complete medium was maintained at 200 µL/well. The culture was progressively expanded from 96-well plates to T25 cell vials, and the fluorescence expression of the cells in each group was observed by fluorescence microscopy. The cells were harvested for qPCR and Western blot, and single-cell clones with accurate identification results were selected for preservation. MDCK cells with knockdown of EGFR were designated sh-EGFR, MDCK cells with overexpression of EGFR were referred to as lv-EGFR, and negative control MDCK cells were designated sh-nc and lv-nc.

### 2.3. RT-qPCR

Total RNA was extracted from cells utilizing TRIzol reagent (10296028CN, Thermo, Waltham, MA, USA). cDNA was synthesized via reverse transcription of RNA according to the guidelines for the HiScript III 1st-Strand cDNA Synthesis Kit (+gDNA wiper) (#R312, Vazyme, Nanjing, China). Quantitative polymerase chain reaction (qPCR) was conducted using the SYBR Green I chimeric fluorescence method. A qPCR system comprising 50 ng of cDNA template, primers, ChamQ Universal SYBR qPCR Master Mix (#Q711, Vazyme, Nanjing, China) and ddH_2_O was established. The reaction conditions were as follows: predenaturation at 95 °C for 30 s; 40 cycles of 95 °C for 10 s and 60 °C for 30 s; reaction at 95 °C for 15 s, reaction at 60 °C for 60 s, and dissolution at 95 °C for 15 s. The experiment was repeated 3 times for each group, and the relative expression of each gene was calculated utilizing the 2^−ΔΔCT^ method based on the CT value of the internal reference gene *GAPDH*. The primer sequences of each gene in this research are shown in Tables S1 and S3.

### 2.4. Western Blot

After reaching the logarithmic growth phase, the culture medium was removed, and the cells were meticulously cleaned three times with cold PBS. The cellular proteins were extracted utilizing a blend of Protein Phosphatase Inhibitor (#P1260, Solarbio, Beijing, China), RIPA buffer (#R0010, Solarbio, Beijing, China), and PMSF buffer (#P0100, Solarbio, Beijing, China) (*V*:*V*:*V* = 1:1:100). The cells were lysed at −20 °C and thawed at room temperature, and the lysis process was repeated multiple times. Centrifugation was performed at 4 °C and 12,000 rpm/min, and 15 min later, the supernatant was extracted. Quantification was performed with the aid of a BCA Protein Quantification Kit (Vazyme, Nanjing, China) to attain a protein concentration of 1 µg/μL. The protein was then mixed with 4× loading buffer (#P1015, Solarbio, Beijing, China) (*V*:*V* = 3:1) and boiled at 95 °C for 15 min. Subsequently, 10 μL of the protein sample was added for acrylamide gel electrophoresis at 80 V for 20 min and at 120 V for 1 h. The proteins were electronically transferred to PVDF membranes (0.45 μm, Merck KGaA, Darmstadt, Germany) with a current of 350 mA for 1 h, and the necessary strips were cut, sealed in 5% skim milk powder for 2 h, and subjected to three 5 min washes with TBST. The membranes were incubated with the primary antibody overnight. On the second day, the membrane was subjected to three 5 min washes with TBST, and then incubated with the secondary antibody (Beyotime, Beijing, China, #A0208, 1:2000) for 1 h and subjected to three 5 min washes with TBST. Superstar ECL plus substrate (Boster Biological Technology, Wuhan, China, #AR1197) was added to the PVDF membrane, which was allowed to react for 1 min, and the exposure time was set to 2 s. Images were collected, and the image data were analyzed using ImageJ (V1.8.0) [16]. The antibodies and dilution ratio were as follows: anti-EGFR (#AF6042, 1:1000), anti-CDK4 (#DF6102, 1:1000), anti-JUK1/2/3 (#AF6318, 1:1500), anti-phospho-JUK1/2/3 (Thr183+Tyr185) (#AF3318, 1:1500), anti-GNB2 (#DF7783,1:1000), anti-GAPDH (#AF7021,1:1000) from Affinity Biosciences, Santa Barbara, CA, USA; Anti-AKT (#9272, 1:1000), anti-phospho-AKT (Ser473) (#4060, 1:1000), anti-MAPK (ERK1/2) (#4695, 1:1000), anti-phospho-MAPK (ERK1/2) (#4370, 1:1000), anti-phospho-PI3 Kinase p85 (#17366, 1:1000), and anti-β-actin (#4970, 1:1000) from the Cell Signaling Technology, BSN, Danvers, MA, USA; Anti-PI3 Kinase p110 β (Proteintech, Wuhan, China, 20584-1-AP, 1:2000); and goat anti-rabbit lgG H&L (HRP) (Abcam, Cambridge, UK, #ab205718, 1:2000).

### 2.5. Cell Proliferation Assay

The CCK-8 assay is used to detect cell proliferation. Upon reaching a cell density of 80~85%, the cells were digested using 0.25% trypsin, and the cell suspension density was adjusted to 1 × 10^4^ cells/mL. The cells were subsequently inoculated onto 96-well plates with 200 μL per well and cultured in an incubator containing 5% CO_2_ at 37 °C. At 24, 48, 72, 96, and 120 h after inoculation, Cell Counting Kit-8 (Cat. No.: HY-K0301, MedChemExpress, Monmouth Junction, NJ, USA) was employed to detect the absorbance values of the cells at 450 nm, and cell proliferation curves were plotted. This process was repeated three times for each set of 6 parallel pairs.

### 2.6. Cell Migration Assay

When the cellular density reached 80%~85%, the cells were enzymatically digested with 0.25% trypsin, and the cell suspension density was adjusted to 3 × 10^4^ cells/mL. Next, 200 μL of the cell suspension was introduced into each well of the ibidi Culture-Insert 2 Well system (ibidi, Martinsried, Germany), placed on a 6-well plate. The external segment of the Culture-Insert was filled with complete medium, and the filling height did not exceed the liquid height within the insert. Three parallel replicates of each experimental group were included in the experiment, and the experiment was repeated 3 times. During the experiment, the cells were cultivated in a 5% CO_2_ incubator at 37 °C. After 24 h of cultivation, once the cellular density in the culture insert reached 100%, as detected under a microscope, the culture insert and the original medium were removed, and 2 mL/well PBS was employed to moisten and dispose of the cells. Then, 2 mL of serum-free DMEM was added, and the cells were cultivated in an incubator. The cell migration capacity was observed and recorded under an inverted microscope at each time point, and the relative migration rate (RMR) of each group of cells in X hours was calculated by:

RMR(%) = (Intercellular area (0 h) − Intercellular area (X h))/(Intercellular area (0 h)) × 100(1)

### 2.7. Colony Formation Assay

Upon reaching a cell density of 80~85%, the cells were enzymatically digested with 0.25% trypsin, and the cell suspension density was adjusted to 1 × 10^4^ cells/mL. A volume of 200 µL of the cell suspension was obtained by adding 9.8 mL of serum-free DMEM to yield a final concentration of 200 cells/mL, and aliquots of 1 mL were added to each well of a 6-well plate. Subsequently, 1 mL of DMEM was added, and the mixtures were thoroughly mixed. The plates were then cultured in a 5% CO_2_ incubator at 37 °C. After 6 days, the 6-well plates were removed from the incubator, the medium was aspirated, and the cells were washed thrice with PBS. Subsequently, the cells were fixed with 1 mL of paraformaldehyde fixative for 15 min. After disposal of the fixing solution, a single wash with PBS was performed, the cells were dyed with crystal violet for 30 min, and the dyeing solution was slowly cleaned under running water to dryness. Each group was processed three times, and three parallel cells of each group were included; the number of clones was counted using ImageJ (V1.8.0) [16] and analyzed statistically.

### 2.8. In Vivo Evaluation of Tumorigenesis

A total of 36 female BALB/c nude mice (the strain name is CAnN.Cg-*Foxn1^nu^*/Crl) aged 28 to 35 days were procured from Beijing Vital River Laboratory Animal Technology Co., Ltd. (Beijing, China) and maintained within specific pathogen-free (SPF) animal facilities. The animals were randomized into 6 groups, given unrestricted access to food and water, and maintained under room temperature at 25 ± 2 °C with cycles of 12 h of light and 12 h of darkness for a pre-experimental adaptation phase of 3 days.

The animal experiment was conducted in compliance with the ARRIVE guidelines and in accordance with the UK Animals (Scientific Procedures) Act (1986) and associated guidelines, EU Directive 2010/63/EU for animal experiments, and the National Research Council’s Guide for the Care and Use of Laboratory Animals. The current animal experimentation underwent rigorous scrutiny and received endorsement from the Experimental Animal Ethics Committee of Northwest Minzu University under approval record number xbmu-sm-2022022.

The cells were cultivated in T175 cell culture vials. When the cell density approached 80~85%, the cells were digested with 0.25% trypsin, and after termination of digestion, the resulting suspension was centrifuged. The cell density of the resulting supernatants was adjusted with aseptic PBS solution to 5 × 10^7^ cells/mL; subsequently, 200 μL of the cell suspension containing 10^7^ viable cells was injected into the left scapula of specific nude mice in the experimental cohort. Meanwhile, 10^6^ HeLa cells were utilized as the positive control, and the negative control consisted of 10^7^ MRC-5 cells. Post-injection, all subjects were meticulously monitored over a protracted duration of 16 weeks. Throughout this period, weekly examinations for clinical symptoms were performed, and during these examinations, palpations were conducted for the identification of nodule growth at the site of inoculation (SOI).

### 2.9. Sensitivity Evaluation of Influenza Virus

The sh-EGFR, lv-EGFR, and their respective control cells were cultured in T25 cell culture vials, and when the cell density approached 80% to 85%, influenza virus was inoculated into the cells according to a multiplicity of infection (MOI) of 0.01. Triplicate parallel experiments were conducted for each cell sample, and the samples were maintained at 34 ± 0.5 °C with 5% CO_2_. The viruses were collected at 48 and 72 h post-inoculation (hpi) and stored at −80 °C.

In a 96-well plate, 1.0 × 10^4^ MDCK cells were inoculated per well to establish a dense monolayer within 24 h. The harvested virus solution was subjected to serial 10-fold dilutions in cell maintenance liquid (DMEM medium containing 2 μg/mL TPCK trypsin) across a gradient of nine concentrations (10^−1^ to 10^−9^), with each diluted virus solution (100 μL) added to individual wells of the plate. Each dilution concentration was replicated six times. Subsequently, the plate was incubated at 34 °C with 5% CO_2_ for complete viral adhesion over a period of 1~2 h, followed by addition of virus maintenance liquid and further incubation for an additional 72 h. Post-incubation, the plate underwent PBS washing, fixation with 4% paraformaldehyde for half an hour, and staining with 0.1% crystal violet. Finally, TCID50 of the viral samples was calculated using the Reed and Muench method [17]. The virus utilized in this research is classified as A/California/7/2009 X-179A (H1N1).

### 2.10. GST Pull-Down Assays

Primers were designed and synthesized based on the specific exons of the *EGFR* gene (Table S2). Splicing by overlap extension polymerase chain reaction (SOE PCR) was employed to amplify the target gene fragment. The pGEX-6P-1 vector bore a *gst* tag, which functions as a reporter protein. The specially engineered target gene and the pGEX-6P-1 vector were separately cleaved by *Bam H*I and *Xho*I restriction enzymes, which resulted in compatible cohesive ends. These blunted regions of the vector and gene were subsequently ligated using T4 DNA ligase, yielding the recombinantly engineered pGEX-6P-1-EGFR plasmid. Sequencing analysis was performed to validate the authenticity of the evolved pGX-6P-1-EGFR clone, which was then transformed into the BL21 (DE3) cell line via chemical transformation. A single bacterial colony harboring the recombinant plasmid was isolated and propagated in an enriched LB liquid medium containing antibiotics at 37 °C until its optical density (OD600) reached approximately 0.6. Part of the bacterial solution was collected as the control group (not induced), and the remaining bacterial solution was added to an IPTG inducer (final concentration 0.5 mM) and cultured at 37 °C for 3 h. After centrifugation, the bacterial pellets were lysed in 40 μL of 1× loading buffer and then subjected to SDS–PAGE analysis to check for visible protein band formation. After expression of high levels of the fusion protein, the bacterial biomass was ultrasonically disrupted, and supernatants and precipitates were isolated for additional SDS–PAGE analyses. The EGFR–GST fusion protein was obtained by purification of the inclusion body or supernatant proteins.

For GST pull-down assays, the EGFR–GST fusion protein (0.5 mg) was incorporated into the treated Mag-Beads GST Fusion Protein Purification (Sangon Biotech, Shanghai, China) and agitated via inversion. The sample was incubated for 30 min at ambient temperature and then washed thrice with wash buffer. Subsequently, total proteins of the MDCK cells (5 mg) were added, and the resulting mixture was incubated at 4 °C overnight. After five washes with wash buffer, proteins were eluted with wash buffer supplemented with 15 mM reduced glutathione. The resultant eluate was fractionated through 12% SDS–PAGE, transferred onto PVDF membranes (distributable from Millipore, Billerica, MA, USA), and exposed to anti-GST antibodies (lot number CSB-MA000304, procured from CUSABIO, Wuhan, China). GST-tagged fusion protein from Wuhan Genecreate (Wuhan, China) was used as a negative control; total proteins of the MDCK cells were used as input. Three replicates of each GST pull-down assay were performed. The proteins present in the elution solutions of both the experimental and control groups were subjected to trypsin hydrolysis, followed by analysis of the resulting peptide fragments using Q Exactive™ HF (Thermo, Waltham, MA, USA) and Ultimate™ 3000 RSLCnano (Thermo, Waltham, MA, USA) mass spectrometry (MS).

### 2.11. Bioinformatic Analysis of Mass Spectrometry (MS) Data

The MS data were compared with the proteome reference database by MaxQuant (V1.6.6) software [18], employing the Andromeda database search algorithm. The reference proteome database for Canis_lupus_familiaris (domestic dog) was obtained from the UniProt database (https://www.uniprot.org/). Initially, the MS data were imported and designated as belonging to either the experimental group or control group. Subsequently, in the “Group-specific parameters” interface, we selected “Modification” options including “Carbamidomethylation (C)”, “Oxidation (M)”, and “Acetylation (Protein N-terminus)”. “Digestion” was set to “Specific” with “Trypsin/P”. The search results were filtered at the protein and peptide segment level using a 1% false discovery rate (FDR) threshold. Proteins absent from the database, contaminated proteins, and those characterized by a single modified peptide segment were excluded; the remaining identification data were subsequently utilized for further analysis. The fold change (FC) ≥ 1.5 and *p*-value ≤ 0.05 were then used as the standard to screen proteins with significant differences. The significantly differentially accumulated proteins were subjected to Gene Ontology (GO) (http://geneontology.org) [19] and Kyoto Encyclopedia of Genes and Genomes (KEGG) (https://www.kegg.jp) analyses [20].

### 2.12. Statistical Analysis

The compilation and graphical representation of the data were executed using the Prism 8.0 software (GraphPad, La Jolla, CA, USA). *p* values were derived from unpaired *t* tests to assess the differences between two groups with normally distributed data. For multigroup comparisons, *p* values were derived by ordinary one-way ANOVA. In all comparisons, *p* < 0.05 (two-sided) was considered to indicate a significant difference (*); *p* < 0.01 (two-sided) was considered to indicate a highly significant difference (**, ***, and ****); and *p* ≥ 0.05 (two-sided) was considered to indicate no significant difference (ns).

## 3. Results

### 3.1. Relation of Tumorigenic Gene Expression in MDCK Cell Lines from Various Origins

Previous investigations have demonstrated that *EGFR*, *APC*, *Huwe1*, and *CUL3* may play a crucial role in the tumorigenicity of MDCK cells, and *JUN* and *MYC* may be intimately related to the proliferation of MDCK cells [13]. Consequently, in this study, *EGFR*, *APC*, *Huwe1*, and *CUL3*, which may be associated with tumorigenicity, were selected, and their relative expression in the MDCK working bank cells (MDCK-WBC) and monoclonal cell lines (MDCK-CA005, MDCK-CB057, MDCK-CL09) was evaluated. The MDCK-WBC cells were tumorigenic (100%), whereas the tumor formation rates of the MDCK-CA005, MDCK-CB057, and MDCK-CL09 cells were 0%, respectively.

**Figure 1:**
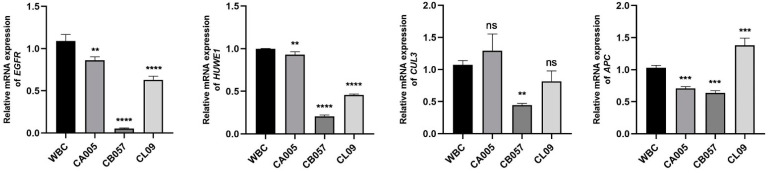
Tumorigenic gene expression in MDCK cell lines from various origins. **, ***, and **** were considered to indicate a highly significant difference (*p* < 0.01); and ns was considered to indicate no significant difference (*p* ≥ 0.05).

Therefore, we employed RT-qPCR to examine the correlation between cellular tumorigenicity and the relative expression levels of these genes. The results shown in Figure 1 indicate that the expression of *EGFR*, *APC*, *Huwe1*, and *CUL3* was detected in four MDCK cell lines from different origins, and the expression level of *EGFR* markedly differed between the bank cells and MDCK-CA005, MDCK-CB057, and MDCK-CL09 cells. Comparable experimental results were also obtained for the *Huwe1* gene. However, the relative expression levels of *CUL3* in MDCK-CA005 and MDCK-CL09 cells did not show significant differences compared to their parental cells, indicating that *CUL3* may not play a pivotal role in regulating cell neoplasia. Similarly, the relative expression level of the gene *APC* in the single-clonal cells exhibited inconsistent patterns. Hence, the *EGFR* and *Huwe1* genes with low expression levels in the cloned cell lines necessitate further investigation, and *EGFR* was designated as the key target gene responsible for the tumorigenicity of MDCK cells in this study.

### 3.2. Construction of Monoclonal Cell Lines of Sh-EGFR and Lv-EGFR

Sh-EGFR recombinant lentivirus and lv-EGFR recombinant lentivirus were transfected into MDCK-WBC cells, respectively, and complete medium was added in 16 h later. Puromycin is capable of suppressing the proliferation of uninfected cells and is thus frequently employed as a screening reagent in cell line screening. In this study, cell clonal clusters derived from single cells were selected by single-cell cloning after four generations of puromycin screening.

**Figure 2:**
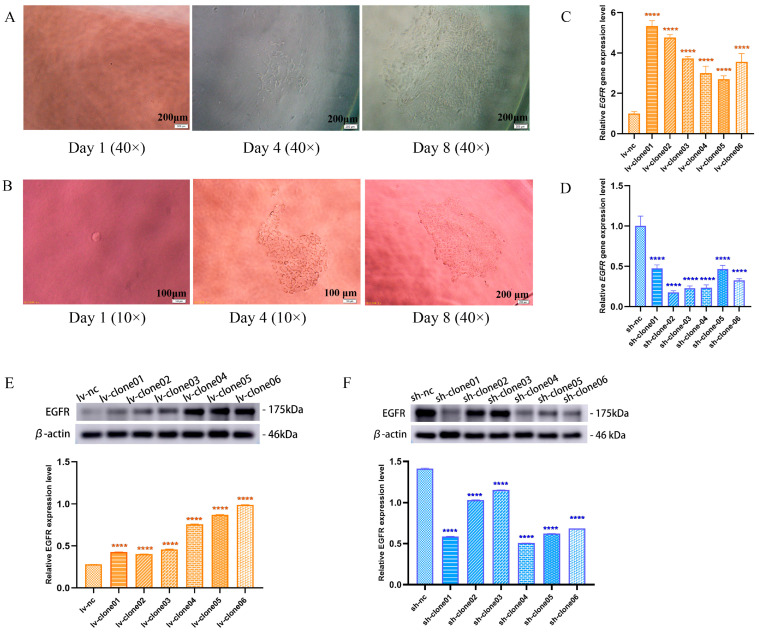
(**A**) Clusters of monoclonal cells at day 1, 4, and 8 after transfection with lv-EGFR lentivirus. (**B**) Clusters of monoclonal cells at day 1, 4, and 8 after transfection with sh-EGFR lentivirus. (**C**) Target gene expression in clone cells with EGFR overexpression. (**D**) Target gene expression in clone cells with EGFR knockdown. (**E**) Western blotting was used to detect the expression bands and relative expression levels of EGFR protein in each lv-clone cell. (**F**) Western blotting was used to detect the expression bands and relative expression levels of EGFR protein in each sh-clone cell. **** was considered to indicate a highly significant difference (*p* < 0.01).

Figure 2A,B depicts the growth progression of single-clone cells transfected with lv-EGFR recombinant lentivirus and sh-EGFR recombinant lentivirus at days 1, 4, and 8. These cells transitioned from individual entities to cell clusters, exhibiting continuous proliferation. Upon reaching T25 culture flasks, distinct cell morphologies emerged in the clonal cell lines as illustrated in Figures S1 and S2. The clone cells were harvested for RT-qPCR and Western blot, and the results demonstrated that despite differing cell morphologies, both clonal cell lines with EGFR overexpression and knockdown effectively exhibit significant difference at the gene or protein level. The relative expression levels of the *EGFR* gene in all overexpression clonal cell lines are summarized in Figure 2C, while the knockdown effects are presented in Figure 2D. Protein level validation results further corroborate these observations (Figure 2E,F). Consequently, we established twelve single-clone cell lines with either EGFR overexpression or knockdown; among these, lv-clone06 and sh-clone04 exhibited superior expression effects and were selected for further investigation under the designations lv-EGFR and sh-EGFR, respectively.

### 3.3. EGFR Can Stimulate the Proliferation, Migration, and Clonogenic Ability of MDCK Cells

**Figure 3:**
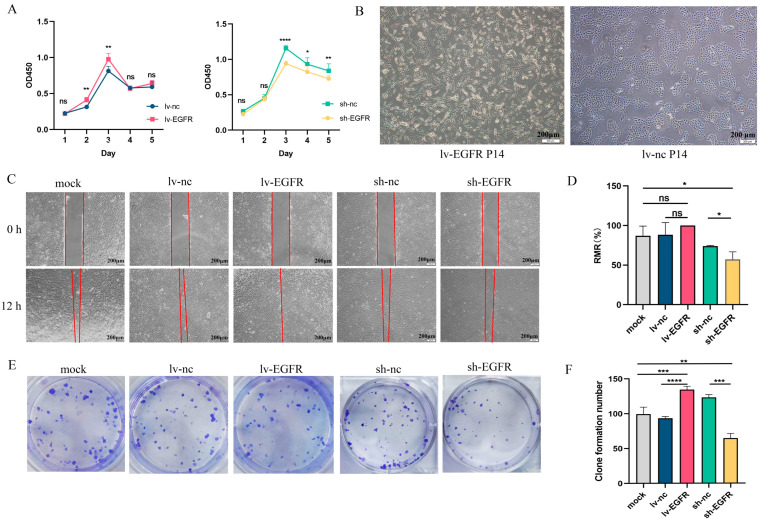
EGFR can stimulate the proliferation, migration, and clonal establishment of MDCK cells. (**A**) The effect of EGFR on cell proliferation was detected by CCK8 assay. (**B**) The morphology of lv-EGFR cells changed after 10 generations. (**C**) Results from the migration assay of each cell group at 0 and 12 h. (**D**) The RMR(%) of each cell at 12 h. (**E**) Graph of the dishes stained for colony formation of cells cultured for 6 days. (**F**) Number of clones formed from each cell group after 6 days of culture. * was considered to indicate a significant difference (*p* < 0.05); **, ***, and **** were considered to indicate a highly significant difference (*p* < 0.01); and ns was considered to indicate no significant difference (*p* ≥ 0.05).

The Cell Counting Kit-8 (CCK-8) assay is currently one of the most widely used methods in life science research for detecting cell proliferation. The CCK-8 reagent can be reduced by dehydrogenase in the mitochondria of cells to generate a highly water-soluble orange-yellow formazan product. The intensity of the color is directly proportional to cell proliferation. Using a microplate reader, the optical density (OD) value at a wavelength of 450 nm is measured, providing an indirect reflection of the number of viable cells. The CCK-8 assay demonstrated that the cells of the experimental and control groups attained their maximum proliferation density at 72 h (3 days) (Figure 3A). The overexpression of EGFR increased the proliferation capacity of MDCK cells, whereas the knockdown of EGFR expression decreased the proliferation capacity of MDCK cells. Furthermore, after 10 generations of culture, the lv-EGFR cells became elongated compared with the control cells and gradually transformed from pebble-like cells to spindle cells (Figure 3B). The migration assay indicated that the overexpression of EGFR could augment the migration capability of MDCK cells, and the RMR reached 100% at 12 h, whereas the relative mobilities of the MDCK-WBC (mock) and lv-nc cells were 86.96% and 88.17%, respectively (Figure 3C,D). The suppression of EGFR expression markedly reduced the migration ability of MDCK cells, and the RMR of sh-EGFR cells at 12 h was merely 57.21%, which was markedly lower than that of the MDCK-WBC (mock) and sh-nc cells. The colony formation assay can evaluate the tumorigenesis capability of cells in the plate. The results demonstrated that the overexpression of EGFR could notably enhance the clonogenic capability of MDCK cells (Figure 3E,F). Five days after the inoculation of 200 cells, the average number of groups with more than 50 clones (clusters d ≥ 0.05 mm) in the plate was 134.3, which was 1.35 times that of the mock group and 1.43 times that of the lv-nc group. Conversely, the clonogenic ability of the MDCK cell lines decreased after the suppression of EGFR expression. Five days after the inoculation of 200 cells, the average number of groups with more than 50 clones (clusters d ≥ 0.05 mm) in the plate was 64.67, which reflected a significantly reduced clonogenic ability compared with that of the mock and sh-nc groups.

### 3.4. EGFR Can Enhance the Tumorigenicity Potential of MDCK Cells In Vivo

**Figure 4:**
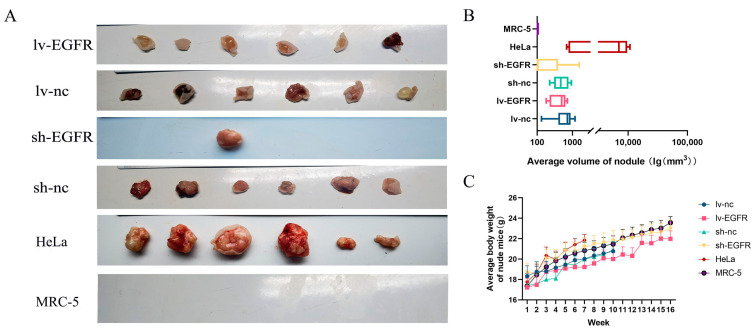
EGFR can enhance the tumorigenicity potential of MDCK cells in vivo. (**A**) Examination of the influence of EGFR on the tumorigenicity of MDCK cells based on xenografts in nude mice. (**B**) Correlation analysis of the mean tumor volumes among experimental groups in concurrent murine tumorigenesis trials. (**C**) Analysis of the trends in the changes in the mean mouse body weight across experimental groups within these mouse trials.

In vivo trials using nude mice indicated that the suppression of EGFR in MDCK cells could restrain tumorigenesis (Table 1 and Figure 4A). Throughout a 16-week animal experiment, nude mice in the HeLa, lv-nc, and sh-nc subgroups were euthanized at weeks 7, 10, and 10 if the nodules exceeded 10 mm in diameter. The results showed that the nodule incidence of all the cohorts, with the exception of the sh-EGFR and MRC-5 groups, was 100%. Specifically, among the sh-EGFR group, a single nude mouse developed a nodule on the posterior and displayed a considerably reduced size compared with its control counterpart (Figure 4B). Furthermore, data on the body weight of the nude mice illustrated that subjects devoid of tumors experienced increased mass gain (Figure 4C).

### 3.5. EGFR Knockdown Increased Influenza Virus Titers in MDCK Cells

Here, we aimed to investigate the impact of EGFR on influenza virus replication within MDCK cells, as understanding the regulatory mechanisms underlying antiviral responses can provide theoretical support for enhancing the production efficiency of cell-based vaccines. The TCID50 method was used to determine the viral titers of different cell-derived virus samples. The TCID50 for each transfected cell was determined using the Reed–Muench method [17].

According to the results presented in Table 2, MDCK cells with overexpressed EGFR exhibited a significant difference in TCID50 compared to control cells at 48 h post-inoculation with the A/California/7/2009 X-179A (H1N1), implying diminished susceptibility of these cells towards the virus. Similarly, the TCID50 obtained from EGFR-overexpressing cells remained inferior compared to that of the control group at 72 hpi. The TCID50 of EGFR knockdown cells was significantly higher than that of control cells 48 h post-inoculation with H1N1 influenza virus. The TCID50/mL can attain a value of 10^−5.60^ at 72 hpi. Our results indicated that both at 48 h and 72 h post-infection, EGFR knockdown cells exhibited significantly higher viral titers. We hypothesize that this phenomenon may be attributed to the activation of innate immune signaling pathways by viral infection, which in turn activates the IFN-β promoter and upregulates antiviral molecules (such as OAS, PKR, and MxA) as well as inflammatory cytokines (including IL-2, IL-6, IFN-α, and IFN-γ). In this context, modulation of EGFR expression—whether downregulated or upregulated—may influence relevant factors within these innate immune signaling pathways.

### 3.6. LC–MS/MS Data Analysis of EGFR-Interacting Proteins

**Figure 5:**
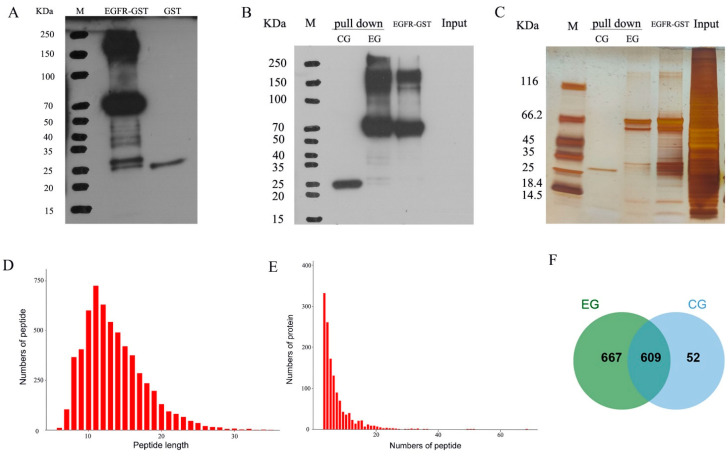
Observations from the GST pull-down assay and subsequent mass spectrometric data evaluation. (**A**) The concentration of GST antibodies in the protein sample was determined by Western blotting prior to conducting the GST pull-down test. M: marker; EGFR–GST: EGFR–GST fusion protein synthesized via an inclusion body, a protein band was detected at approximately 66 kDa; GST: GST protein, a protein band was detected at ~25 kDa. (**B**) The expression of GST antibody in protein was detected by Western blotting after the pull-down test. M: marker; pull-down-CG: protein bands evident within the control group’s eluate, which displayed the previously identified ~25-kDa band; pull-down-EG: specific protein bands observed in the test group’s eluate, which corresponded to the preestablished ~66-kDa EGFR–GST protein; input: total protein content of the MDCK cell line employed in the study. (**C**) The protein expression in each group was detected by silver staining after the pull-down test. As noted earlier, this group mirrors those depicted in panel B. (**D**) Number of peptides of different lengths detected by MS. The horizontal coordinate is the number of amino acids that compose the peptide segment, namely, the length of the peptide segment, and the vertical coordinate is the number of peptide segments. (**E**) Number of proteins with different peptide numbers detected by MS. The horizontal coordinate is the number of peptides that make up the protein, and the vertical coordinate is the number of proteins. (**F**) Venn diagram visualizing the interacting protein network derived by MS analysis of the test versus control groups.

The molecular size of the EGFR–GST fusion protein was estimated to equal 66 kDa. The fusion protein mainly existed in the form of inclusion bodies, as revealed by SDS–PAGE of the supernatant and precipitates of the crushed bacteria. After the expression of many proteins, the EGFR–GST fusion protein was obtained through purification and renaturation (Figure S1). EGFR–GST or GST tag protein was used as the ‘bait’ protein for the pull-down assay. The experimental group (designated EG) consisted of EGFR–GST protein, Sepharose beads, and total protein from MDCK cells. The control group (designated CG) contained GST tag protein, Sepharose beads, and total protein from MDCK cells. Western blot and silver staining tests were then performed using the EG and CG protein eluents. The results showed that the expression of the target protein EGFR–GST was detected in the EG, whereas no target protein was detected in the CG, and different bands were detected between the experimental group and the control group (EGFR–GST) (illustrated in Figure 5A–C). The eluate samples were tested by LC–MS/MS.

The majority of the peptides quantified by MS (99.15%) encompassed between seven and twenty-seven amino acids. These data are clearly depicted in Figure 5D. Significantly, 75.09% of the explored proteins exhibited at least two identifiable peptides, as illustrated in Figure 5E. The proportion of non-missing peptides was 88.55%, and the proportion of contaminated proteins was 2.39%. In this study, the proteins obtained from the EGFR–GST fusion protein pull-down test and GST protein pull-down test were analyzed by MS, leading to the identification of a comprehensive set of 1328 proteins. Notably, some unique results surfaced: 52 proteins were only found in the CG, 667 proteins were exclusively observed in the E group, and 609 proteins were detected in both groups (Figure 5F). To focus on proteins other than GST proteins, we conducted extensive functional evaluations of the 667 EGFR-interacting proteins found exclusively in the EG.

Gene Ontology (GO) annotation analysis effectively elucidates the molecular function (MF), cellular component (CC), and biological process (BP) terms of the genes of interest. Consequently, a GO annotation analysis of 667 EGFR-interacting proteins in the EG microenvironment was conducted. The findings indicated that the primary GO annotations within the BP category encompassed ‘regulation of cellular process’ (GO:0050794), ‘macromolecule metabolic process’ (GO:0043170), ‘organonitrogen compound metabolic process’ (GO:1901564), and ‘cellular component organization’ (GO:0016043). The majority of interacting proteins were enriched in specific cellular subregions, notably ‘membrane-bounded organelle’ (GO:0043227) and ‘intracellular membrane-bounded organelle’ (GO:0043231) in the CC. Concurrently, the MF categories were strongly correlated with terms such as ‘enzyme binding’ (GO:0019899), ‘nucleic acid binding’ (GO:0003676), and ‘identical protein binding’ (GO:0042802). Thus, our observations suggest that the identified interacting proteins play diverse biological roles within distinct cellular regions and thereby substantially contribute to cell metabolism.

**Figure 6:**
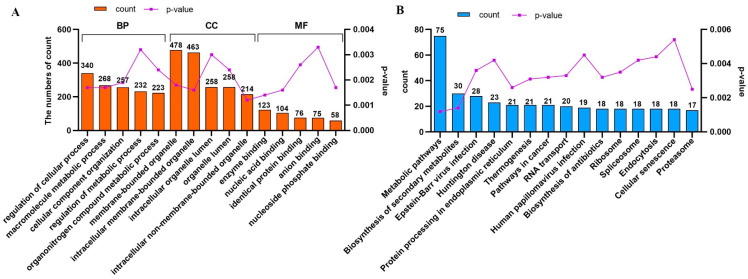
Comprehensive Gene Ontology (GO) and Kyoto Encyclopedia of Genes and Genomes (KEGG) analyses of interacting proteins. (**A**) The details related to the top five terms in the ‘Biological Process’, ‘Cellular Component’, and ‘Molecular Function’ categories found for the interacting proteins in the GO analysis are presented. (**B**) The biological pathways in which the interacting genes (proteins) are mapped onto the top 15 positions within a specifically defined pathway.

The sequential mapping of EGFR-associated molecular entities revealed their conceptualization within 296 indices attributed to relevant pathways. Figure 6 illustrates the 15 main pathways with the highest degree of enrichment, which included cellular phenomena linked to metabolism, biosynthesis, and protein manipulation. This finding underscores the pivotal position of EGFR in orchestrating varied cellular functions across numerous molecular channels. Notably, ‘pathway in cancer’ included 21 associated proteins, representing a compelling argument for the regulatory control exerted by EGFR during MDCK cell tumor initiation. Table 3 provides comprehensive details regarding these 21 interacting proteins.

### 3.7. The EGFR Can Activate the PI3K–AKT Signaling Pathway

**Figure 7:**
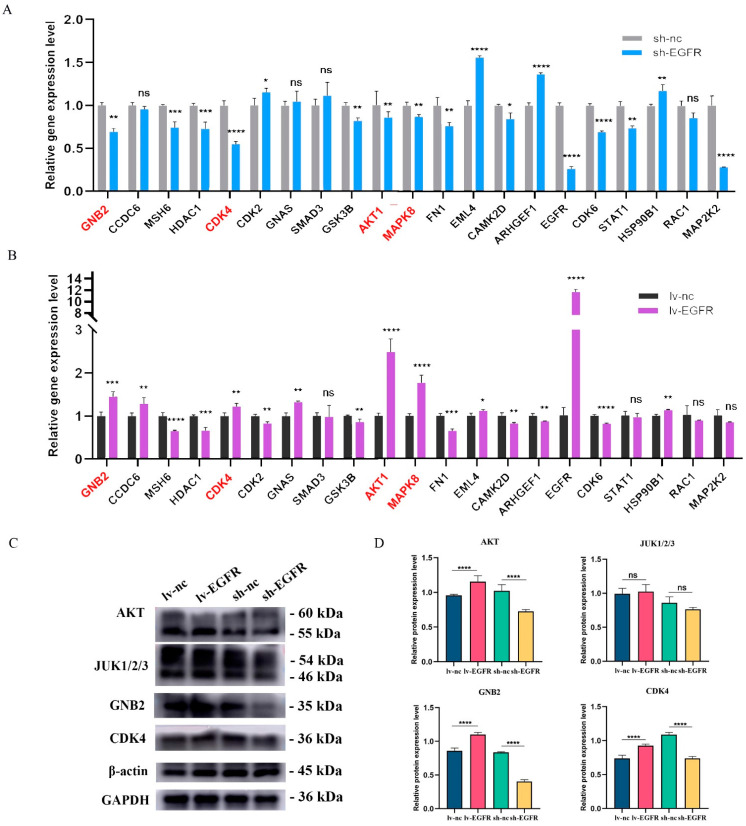
Validation of genes and proteins interacting with EGFR. (**A**) The relative gene expression levels of 21 interacting proteins in EGFR knockdown cells and its control cells. (**B**) The relative gene expression levels of 21 interacting proteins in EGFR overexpression cells and its control cells. (**C**) Protein levels were detected by screening interacting proteins in different cell groups. (**D**) Histogram of quantized analysis of interacting proteins in different cell groups. * was considered to indicate a significant difference (*p* < 0.05); **, ***, and **** were considered to indicate a highly significant difference (*p* < 0.01); and ns was considered to indicate no significant difference (*p* ≥ 0.05).

We utilized the RT-qPCR method to verify the expression of 21 genes identified by MS in MDCK cells with overexpressed and interfered EGFR. The results are presented in Figure 7. The relative gene expression levels of four genes, *AKT1*, *CDK4*, *GNB2*, and *MAPK8*, significantly decreased in EGFR knockdown cells (Figure 7A), while they significantly increased in MDCK cells with overexpressed EGFR (Figure 7B). Therefore, AKT1, CDK4, GNB2, and MAPK8 may have an interaction relationship with EGFR. Subsequently, we respectively detected the correlations between EGFR knockdown and overexpression and the four genes *AKT1*, *CDK4*, *GNB2*, and *MAPK8* (*JUN1/2/3*) at the protein level. The results of Western blot showed (Figure 7C,D) that the expression levels of GNB2, AKT, and CDK4 were significantly downregulated in EGFR knockdown cells, while significantly upregulated after EGFR overexpression; MAPK8 (JUN1/2/3) did not show changes in expression levels with EGFR knockdown or overexpression.

**Figure 8:**
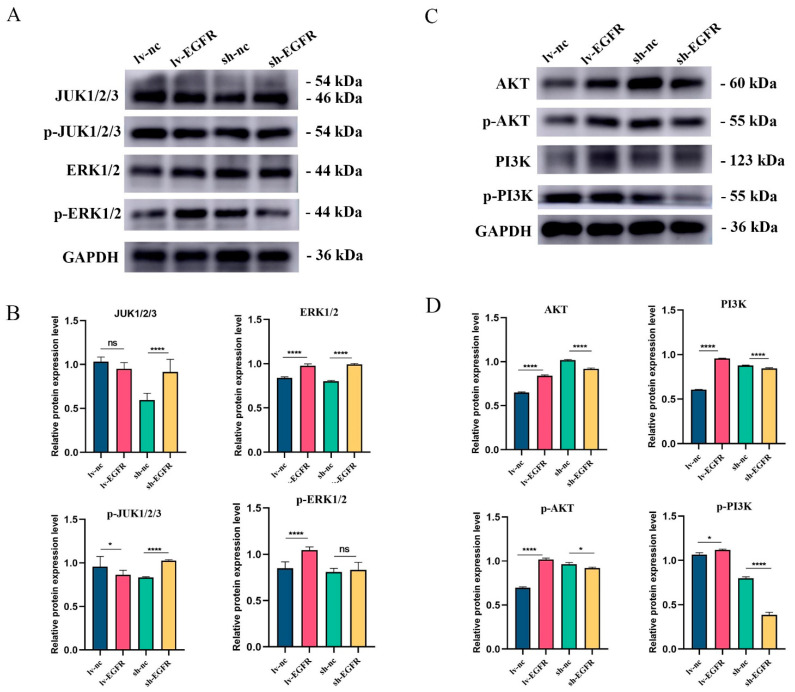
Protein expression and phosphorylation degree of key factors in MAPK–ERK and PI3K–AKT signaling pathway. (**A**) Western blot analysis of JUK1/2/3, ERK1/2, and their phosphorylation levels in different cell groups. (**B**) Histogram of quantized analysis of each strip in the MAPK–ERK signal path. (**C**) Western blot analysis of AKT, PI3K, and their phosphorylation levels in different cell groups. (**D**) Histogram of quantized analysis of each strip in the PI3K–AKT signal path. * was considered to indicate a significant difference (*p* < 0.05); **** was considered to indicate a highly significant difference (*p* < 0.01); and ns was considered to indicate no significant difference (*p* ≥ 0.05).

Given that AKT1 and MAPK8 are pivotal molecules in the PI3K–AKT and MAPK–ERK signaling pathways, respectively, and EGFR, a tyrosine kinase, can transmit multiple signaling pathways and stimulate the activation and phosphorylation of downstream signaling molecules, we assessed the total protein and phosphorylated protein levels of related molecules in the PI3K–AKT and MAPK–ERK signaling pathways to validate the signaling pathways linked to EGFR expression. The pertinent research outcomes are illustrated in Figure 8. Following EGFR overexpression or knockdown, the proteins JUK1/2/3 and ERK1/2 in the MAPK–ERK signaling pathway did not exhibit significant increase or decrease, and no correlation was observed between the phosphorylation degrees of p-JUK1/2/3 and p-ERK1/2 and their total proteins. However, upon EGFR overexpression, when examining the expression of key molecules in the PI3K–AKT signaling pathway in lv-EGFR and its control cells, it was discovered that the protein expressions of AKT and PI3K exhibited a significant increase, and the phosphorylated proteins p-AKT and p-PI3K dramatically increased, signifying that the overexpression of EGFR activated the PI3K–AKT signaling pathway, leading to the phosphorylation of AKT and PI3K proteins. Conversely, following the suppression of EGFR protein expression in cells, the protein expressions of AKT and PI3K demonstrated a significant decrease, and the PI3K–AKT signaling pathway was inhibited.

## 4. Discussion

Previous research has revealed that the tumorigenicity of MDCK cells appears to be closely associated with members of the ErbB family of RTKs [13,14]. Initially, we conducted a comprehensive analysis of the gene expression profile of MDCK cells sourced from various sources, including cell lines in the ATCC and certain monoclonal cell lines. As expected, our findings indicated that the EGFR gene exhibited significantly lower expression levels in monoclonal cell lines devoid of tumorigenicity, whereas its expression levels were considerably elevated in the MDCK cell line from ATCC. This evidence underscores the widespread existence of cellular heterogeneity. Subsequently, we successfully established MDCK cell lines with stable knockdown or overexpression of the EGFR protein. Our experiments revealed that EGFR was capable of potentiating cell proliferation, migration, and colony formation. The results of tumorigenicity assays conducted using nude mice demonstrated that EGFR played a pivotal role in facilitating the onset of tumorigenesis in vivo. Studies have shown that normal cells have strict requirements for survival and proliferation, while cells expressing oncogenes exhibit growth characteristics similar to those of tumor-isolated cells but different from those of normal cells [21,22]. For example, although normally cultured fibroblasts exhibit contact inhibition, they require growth anchoring, depend on the serum in the growth medium, and exhibit a limited replicative lifespan; however, oncogene-transformed cells exhibit resistance to contact inhibition, display the ability to form lesions, proliferate in semisolid media, have a reduced requirement for serum, and possess the capacity for indefinite growth [23]. In the aforementioned review, we highlight the correlation between tumorigenicity in MDCK cells and cellular heterogeneity as well as the notion of epithelial–mesenchymal transition (EMT) [10]. The inherent heterogeneity of the MDCK cell population establishes an intracellular milieu that closely mimics the tumor microenvironment (TME), which is characterized by the presence of distinct cellular subsets harboring stem cell subpopulations expressing tumorigenic phenotypes. Each subpopulation had the capacity to form tumors in vivo, was retained throughout successive passages, and exhibited divergent susceptibilities to hormonal and other stimulatory stimuli. Consequently, a population of cells exhibiting either tumorigenic or non-tumorigenic characteristics was generated [6]. Epithelial cells acquire the characteristics of invasiveness and tumorigenicity through the induction of EMT. During this process, epithelial cells progressively lose their distinctive pebble-like cell appearance in monolayer culture and gradually transform into spindle-shaped mesenchymal cells [6,24,25]. In this study, we found that cells overexpressing EGFR showed corresponding changes in their high proliferation rate. After passage, these cells assumed a smaller, elongated, and more refractive phenotype (Figure 3), whereas the cells with suppressed EGFR expression exhibited diminished growth. Similarly, investigations have demonstrated that both bFGF and PDGD can foster the anchorage-independent growth of diploid fibroblasts, and PDGF-D even promotes the malignant transformation of NIH3T3 fibroblasts, resulting in a propensity for soft agar cloning and tumorigenesis in nude mice [26,27,28].

EGFR, a member of the ErbB family of RTKs, plays a pivotal role in the physiology of epithelial cells [29]. The expression and functionality of the *EGFR* gene can be altered through genetic mutations or amplification, which leads to abnormal EGFR signaling and tumorigenesis. Consequently, EGFR overexpression is commonly observed in numerous solid tumors, which makes it an ideal therapeutic target for the treatment of various malignancies in humans [30]. Using a pull-down assay and MS detection, we identified a total of 21 EGFR-interacting proteins that are implicated in tumor formation. Upon validation, EGFR’s interactions with AKT1, CDK4, GNB2, and MAPK8 were confirmed at both genetic and protein levels. G protein subunit β 2 (GNB2) is pivotal in transmembrane conduction, activating G-protein-coupled receptors and downstream effector proteins, and regulating signal transduction pathways. Mutations or overactivation of GNB2 can result in Sturge–Weber syndrome [31]. GNB2 was upregulated in 23 types of cancer, and its increased expression was associated with a lower overall survival (OS) in liver hepatocellular carcinoma (LIHC) and rectal adenocarcinoma (READ) [32]. Yoda et al. reported that diverse GNB2 mutations can activate typical signaling pathways and confer resistance to targeted kinase inhibitors in various cancers, including acute myeloid leukemia and melanoma [33]. CDK4, a member of the cell-cycle-dependent kinase protein family, positively regulates cell cycle regulatory factors, inducing rapid transition from G1 phase to S phase and promoting tumor growth [34]. Further investigation revealed that EGFR can activate key factors of the PI3K–AKT signaling pathway, AKT and PI3K, and promote their phosphorylation levels. Upregulation of EGFR triggers the PI3K-recruitment-based signal cascade, where PI3K catalyzes PIP3 formation. PIP3 binds to the PH domain of AKT and PDK1 (phosphatidylinositol 4,5-bisphosphate delta isomerase), phosphorylating AKT, inhibiting autophagy, blocking GLUT1 endocytosis, and increasing GLUT1 on the cell plasma membrane, thereby accelerating glucose uptake and stimulating cell proliferation [29,35].

Cell culture is influenced by a multitude of factors, including serum and medium components. The medium provides carrier proteins, vital nutrients, hormones, and growth factors, promoting cell adhesion to the plastic surface. We investigate the mechanism of MDCK tumorigenicity, with epidermal growth factor receptor (EGFR) potentially influencing numerous proteins, potentially playing a pivotal role in cell tumorigenicity. Reducing the quantity of growth factors in the medium, removing serum growth factors, or decreasing serum usage may suppress cell tumorigenesis and reduce potential infectious factors in the serum, representing a promising avenue for future research and improved cell substrates for cell-based vaccines.

## 5. Conclusions

The experiments and analysis described in this article provide evidence showing that EGFR plays a pivotal role in the tumorigenicity of MDCK cells, which makes it an effective target for reducing this phenomenon. The *EGFR* gene was expressed at lower levels in non-tumorigenic MDCK cells, but its overexpression could significantly enhance cell proliferation, migration, and clonal formation and augment the tumorigenic potential of these cells in vivo. EGFR knockdown could effectively curtail tumorigenesis and amplify the titers of influenza viruses in MDCK cells. An analysis of the underlying mechanism identified a total of 21 interacting proteins implicated in tumor formation, and among these, AKT1, CDK4, GNB2, and MAPK8 were confirmed at both gene and protein levels. Further investigation revealed that EGFR can activate key factors of the PI3K–AKT signaling pathway, AKT and PI3K, and promote their phosphorylation levels. Consequently, we concluded that EGFR interacts with GNB2, facilitating transmembrane signal transduction, activating the PI3K–AKT signaling cascade, controlling cell cycle alterations, stimulating cell proliferation, and promoting tumorigenesis.

## Figures and Tables

**Table 1:** Results of in vivo tumorigenicity assays.

Cell	Number of Tumor Formation (*n* = 6)	Average Volume of Nodule (mm^3^)	Average Body Weight of Nude Mice (g)
lv-EGFR	6/6	455.8	21.98
lv-nc	6/6	670.3	20.77
sh-EGFR	1/6	258.4	22.97
sh-nc	6/6	522.7	20.92
HeLa (positive control)	6/6	5893	21.85
MRC-5 (negative control)	0/6	0	23.56

**Table 2:** Virus titers TCID_50_/0.1 mL of transfected cell 48 h and 72 h after infection with H1N1.

	LgTCID_50_/mL	Cells			
Times		lv-nc	lv-EGFR	sh-nc	sh-EGFR
48 hpi		5.67	5.50 ***	5.34	5.63 ****
72 hpi		5.60	5.50 **	5.25	5.60 ****

**, ***, and **** were considered to indicate a highly significant difference (*p* < 0.01).

**Table 3:** Interacting proteins mapped to the pathway in cancer in KEGG.

Name	Gene	MW (kDa)	Description
A0A8I3ME07	*GNB2*	37.331	G protein subunit β 2
A0A8I3MLL4	*CCDC6*	48.055	Coiled-coil domain containing 6
A0A8I3MPE7	*MSH6*	137.329	mutS homolog 6
A0A8I3MVP1	*HDAC1*	55.162	Histone deacetylase 1
A0A8I3N1C2	*CDK4*	22.227	Cyclin-dependent kinase 4
A0A8I3N2E6	*CDK2*	27.093	Cyclin-dependent kinase 2
A0A8I3NFU9	*GNAS*	44.899	GNAS complex locus
A0A8I3P2U7	*SMAD3*	46.99	SMAD family member 3
A0A8I3P4F2	*GSK3B*	37.174	Glycogen synthase kinase 3 β
A0A8I3P9C5	*AKT1*	55.756	AKT serine/threonine kinase 1
A0A8I3PEK4	*MAPK8*	19.554	Mitogen-activated protein kinase 8
A0A8I3PLI0	*FN1*	250.347	Fibronectin 1
A0A8I3PSA8	*EML4*	102.573	EMAP like 4
A0A8I3Q089	*CAMK2D*	47.672	Calcium/calmodulin-dependent protein kinase II delta
A0A8I3RRK9	*ARHGEF1*	99.24	Rho guanine nucleotide exchange factor 1
A0A8I3RYJ5	*EGFR*	132.688	Receptor protein–tyrosine kinase
A0A8I3RZY6	*CDK6*	36.976	Cyclin-dependent kinase 6
A0A8I3S5V1	*STAT1*	81.911	Signal transducer and activator of transcription
P41148	*HSP90B1*	92.514	Heat shock protein 90 β family member 1
P62999	*RAC1*	21.45	Ras-related C3 botulinum toxin substrate 1
Q1HG70	*MAP2K2*	44.446	Dual specificity mitogen-activated protein kinase kinase 2

## Data Availability

The original contributions presented in the study are included in the article/Supplementary Material, further inquiries can be directed to the corresponding author.

## Supplementary Materials

The following supporting information can be downloaded at: https://www.mdpi.com/article/10.3390/genes15091208/s1, Figure S1. Vector information and maps of plasmids GV298 and GV492; Figure S2. Monoclonal Cell Lines of lv-EGFR were observed under a fluorescence microscope; Figure S3. Monoclonal Cell Lines of sh-EGFR were observed under a fluorescence microscope; Figure S4. SDS-PAGE analysis of protein expression; Table S1. Primer sequences of tumorigenicity-related factors; Table S2. The primer sequences required for SOE PCR; Table S3. Sequence of gene primers of interacting proteins.
