# Supplementary material for: Epidermal Growth Factor Receptor Emerges as a Viable Target for Reducing Tumorigenicity of MDCK Cells
Source: Genes (Basel). 2024 Sep 14;15(9):1208. doi: 10.3390/genes15091208 (PMC11431121; doi:10.3390/genes15091208)
Supplement: Supplementary file 1 [file genes-15-01208-s001.zip › genes-3189650-supplementary.pdf]

Supplementary Graphic and Tables within the article

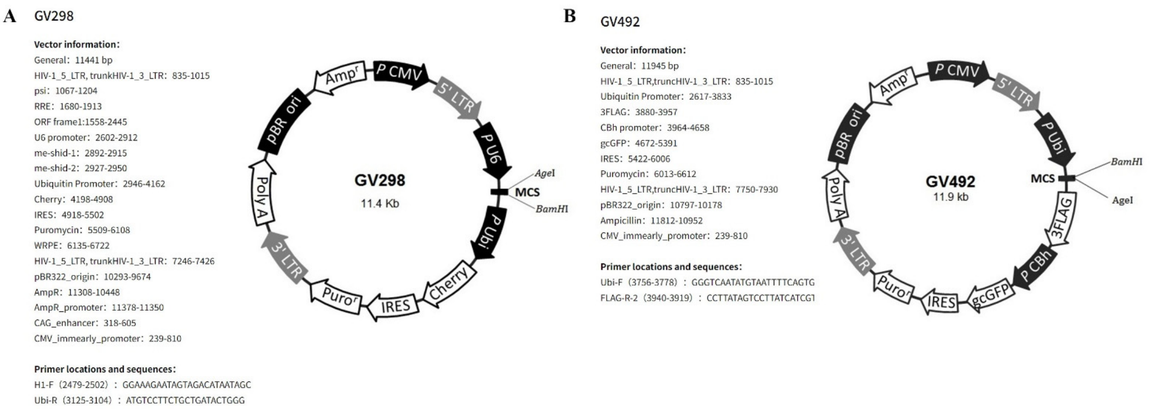

Figure S1. Vector information and maps of plasmids GV298 and GV492. (A) Vector information and maps of plasmid GV298. (B) Vector information and maps of plasmid GV492.

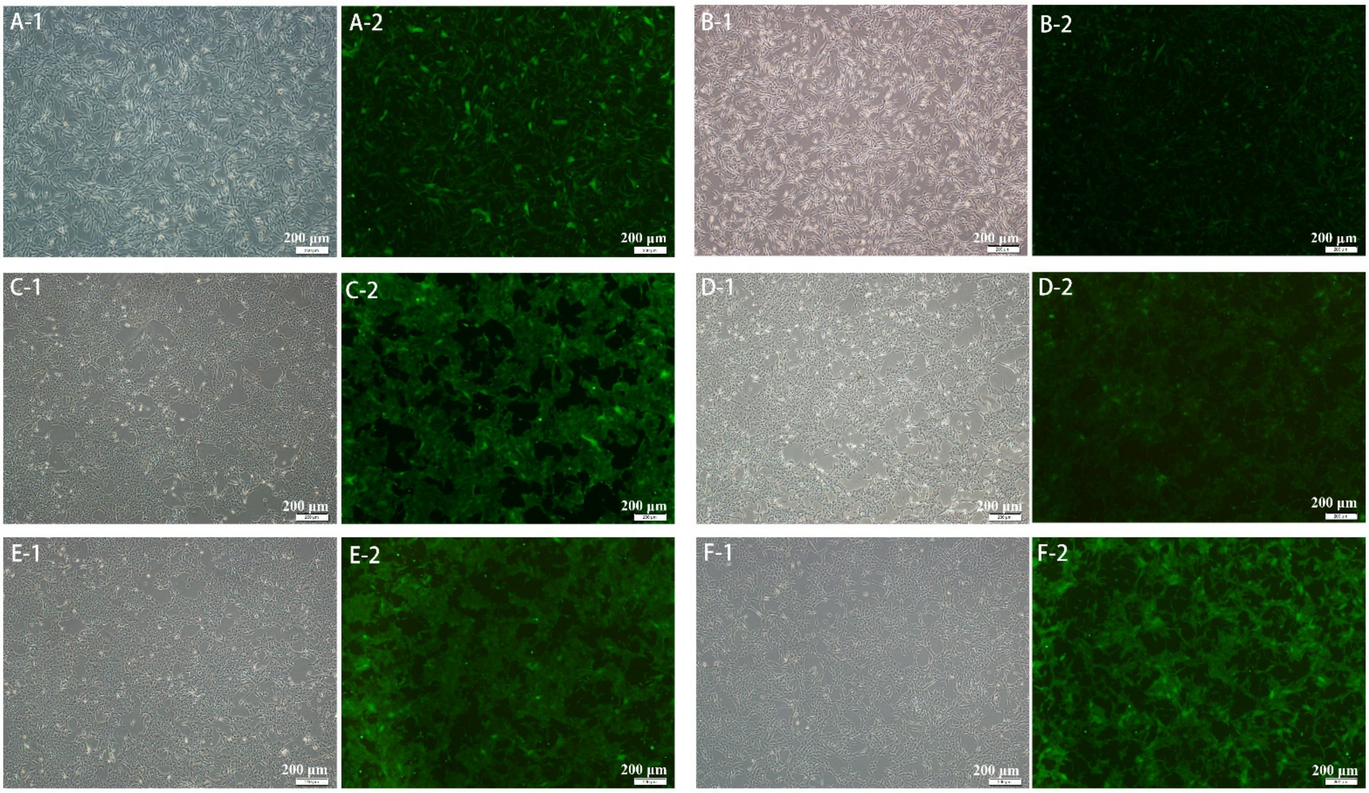

Figure S2. Monoclonal Cell Lines of lv-EGFR were observed under a fluorescence microscope. Photographs A to F showed the cell morphology of lv-clone01 to lv-clone06, respectively. Photographs A1 to F1 showed cells in the bright-field state from lv-clone01 to lv-clone06, respectively. Photographs A2 to F2 showed the lv-clone cells exhibit the GFP green fluorescent protein.

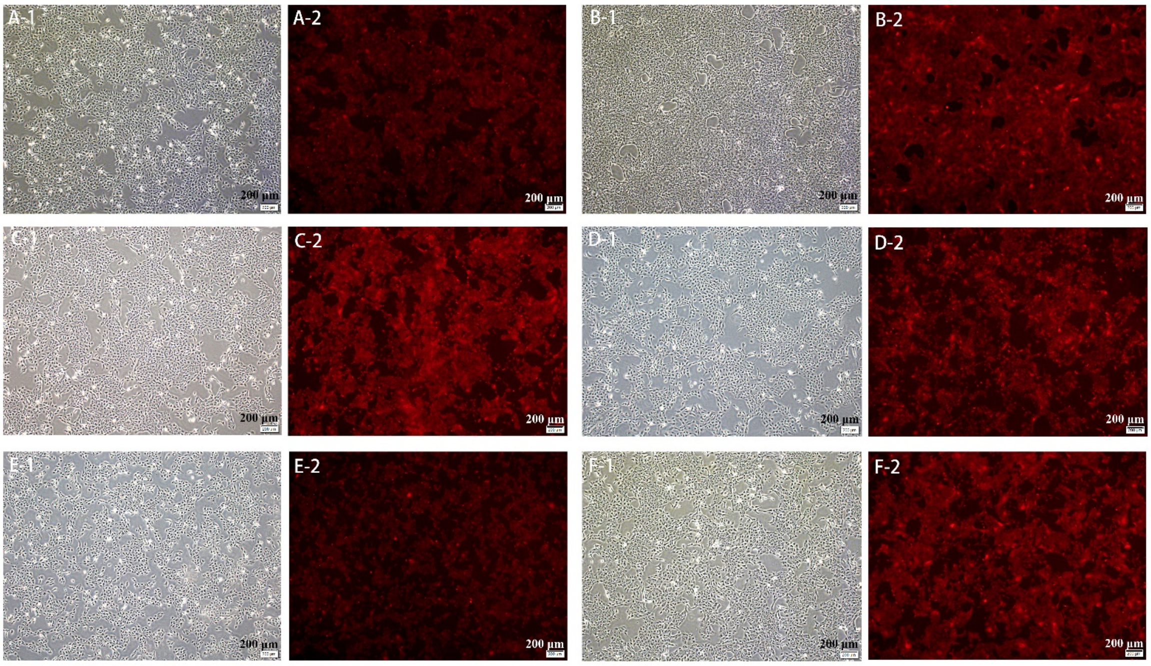

Figure S3. Monoclonal Cell Lines of sh-EGFR were observed under a fluorescence microscope. Photographs A to F showed the cell morphology of sh-clone01 to sh-clone06, respectively. Photographs A1 to F1 showed cells in the bright-field state from sh-clone01 to sh-clone06, respectively. Photographs A2 to F2 showed the sh-clone cells exhibit the Cherry red fluorescent protein.

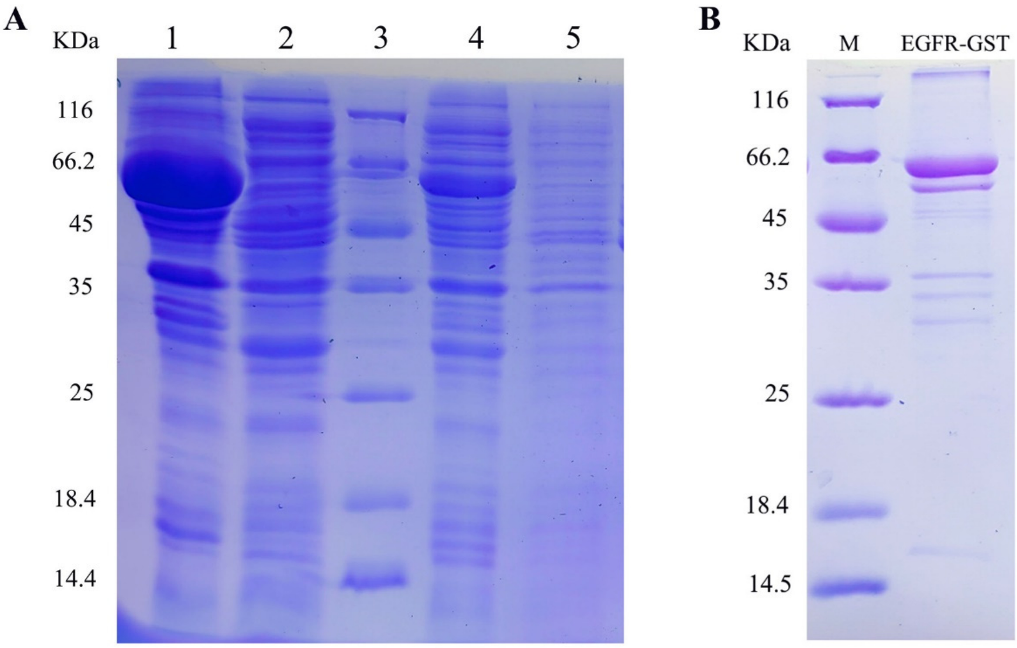

Figure S4. SDS-PAGE analysis of protein expression. (A) *E. coli* BL21 (DE3) after lysis for assessment of fusion protein expression. 1: Bacterial precipitation when the target protein is expressed in large quantities; 2: The bacterial supernatant when the target protein is expressed in large quantities; 3: Protein marker; 4: Bacterial precipitation when the target protein is expressed in small quantities; 5: Untransformed plasmid of BL21 (DE3). (B): EGFR-GST fusion protein was obtained through purification and renaturation. 1: Protein marker; 2: EGFR-GST fusion protein (~66-kDa).

Table S1. Primer sequences of tumorigenicity-related factors.

Primer	Primer sequence
EGFR_Forward	TAATGGAATAGGGATTGGAG
EGFR_Reverse	TAGAGGTAGGGTATGCGTGA
APC_Forward	CCAAACGCTAAAGATGCA
APC_Reverse	CAGGGACAGGGTTACTCG
HUWE1_Forward	CCGCTTCTTTACGAACATTA
HUWE1_Reverse	GTGGCAAACTGGTGAGGATA
CUL3_Forward	TCTAATACACGGAAGCACAT
CUL3_Reverse	GTTTACCACAGGCGAGGGAC
GADPH_Forward	AGTGACACCCACTCTTCCACCT
GADPH_Reverse	GTGGTCCAGGAGGCTCTTACTC

Table S2. The primer sequences required for SOE PCR

No.	Sequence(5'->3')	Length	Position
1	TTCTGTTCCAGGGGCCCCTGGGATCCATGACCCTGCGTCGCCTGCTGCAGGAACGCGAA	59	+:0:58
	TTCGGGGCTTCACCGCTCGGGGTCAGCGGTTCCACCAGTTCGCGTTCCTGCAGCA	55	-:42:96
2	AGCGGTGAAGCCCCGAATCAGGCCCTGCTGCGCATTCTGAAAGAAACCGAATTTAAAAA	59	+:80:138
	TTATAAACGGTACCAAAGGCACCGCTGCCCAGCACCTTGATTTTTTTAAATTCGGTTTC	59	-:122:180
3	TTTGGTACCGTTTATAAAGGTCTGTGGATTCCGGAAGGCGAAAAAGTTAAAATTCCGGT	59	+:164:222
	TTTGCTTTCGGGCTGGTGGCTTCGCGCAGTTCTTTAATTGCCACCGGAATTTTAACTTT	59	-:206:264
4	ACCAGCCCGAAAGCAAATAAAGAAATTCTGGATGAAGCCTATGTTATGGCAAGTGTTGA	59	+:248:306
	CTGGTCAGACAAATGCCCAGCAGGCGGCACACATGCGGATTATCAACACTTGCCATAAC	59	-:290:348
5	GGCATTTGTCTGACCAGCACCGTGCAGCTGATTACCCAGCTGATGCCGTTTGGTTGCCT	59	+:332:390
	TGACTGCCGATATTATCTTTATGTTCACGAACATAATCCAGCAGGCAACCAAACGGCAT	59	-:374:432
6	GATAATATCGGCAGTCAGCATCTGCTGAATTGGTGCGTGCAGATTGCCAAAGGCATGAA	59	+:416:474
	GCGGCCAGATCACGATGCACCAGACGACGATCTTCCAGATAATTCATGCCTTTGGCAAT	59	-:458:516
7	CATCGTGATCTGGCCGCACGCAATGTGCTGGTTAAAACCCCGCAGCATGTTAAAATTAC	59	+:500:558
	TCTTTTTCTTCTGCACCCAGCAGTTTTGCCAGACCAAAATCGGTAATTTTAACATGCTG	59	-:542:600
8	GGTGCAGAAGAAAAAGAATATCATGCCGAAGGTGGTAAAGTTCCGATTAAATGGATGGC	59	+:584:642
	TCGCTCTGATGGGTATAAATGCGATGCAGAATGCTTTCCAGTGCCATCCATTTAATCGG	59	-:626:684

9	TATACCCATCAGAGCGATGTTTGGAGCTATGGTGTGA CCGTGTGGGAACTGATGACCTT	59	+:668:726
	CTAATTTCGCTTGCCGGAATGCCATCATACGGTTTAC TACCAAAGGTCATCAGTTCCCA	59	-:710:768
10	CCGGCAAGCGAAATTAGCACCATTCTGGAAAAAGGT GAACGTCTGCCGCAGCCGCCGAT	59	+:752:810
	AGCACTTAACCATAATCATATACACATCAATGGTACA AATCGGCGGCTGCGGCAG	55	-:794:848
11	GATTATGGTTAAGTGCTGGATGATTGATGCAGATAGC CGTCCGAAATTTCGTGAACTGA	59	+:832:890
	ATAGCGCTGCGGATCGCGTGCCATCTTGCTAAATTCA ATAATCAGTTCACGAAATTTC	58	-:874:931
12	GCGATCCGCAGCGCTATCTGGTGATTCAGGGCGATGA ACGCATGCATCTGCCGAGTCCG	59	+:915:973
	TCTTCTTCATCCATCAGGGCACGATAAAAATTACTAT CGGTCGGACTCGGCAGATGCA	58	-:957:1014
13	CTGATGGATGAAGAAGATATGGAAGATGTGGTGGAT GCAGATGAATATCTGATTCCGCA	59	+:998:1056
	AGTCAGTCACGATGCGGCCGCTCGAGTTAAAAAAA CCCTGCTGCGGAATCAGATATTC	59	-:1040:1098

Table S3. Sequence of gene primers of interacting proteins.

Primer	Primer sequence	Primer	Primer sequence
GNB2	GCGGAGATGTGATGTCCCTAT<br>CACGGCATTGATGTCTGACTC	MAPK8	CCACCACCAAAGATACCTGACA<br>TGACGACGATGGATGCTGAG
CCDC6	GGATGACGAAAGGTATTTTAACGAG<br>GCTTGAACTTGGCGAAGGTGT	FN1	AGGCTACTATCACTGGTCTGGAAC<br>TCTTTTGAACTGTGGAGGGAAC
MSH6	GGAAGGTCTCATACCAGGCTCC<br>CATCACCCCACTGTCCTCATT	EML4	AGGAAGGTGATTGCTGTCGC<br>GCTGTGGGCACTGTACTTGTGA
HDAC1	GGAGATGTTCCAGCCCAGTG<br>TGCCCTTTGATCGTGAGATTG	CAMK2D	ACCCTGCCAAGCGTATCAC<br>GCATCATGGAAGCAACAGTAGAA
CDK4	GCTGAGATTGGGGTTGGTG<br>CGAACTGTGCTGATGGGAAG	ARHGEF1	CCTCGGTTCTGTGCCTTTG<br>CTTTCTCCCGTTCTGCTGACT
CDK2	ATGGATGCTTCTGCTTTGACTG<br>GAGGTTTGAGGTCTCGGTGC	CDK6	TGGACCTCTGGAGTGTTGGC<br>ATCGGTTGAGGAGATTTTGAGTG
GNAS	ATGAACGCCGCAAATGG<br>CTGGTTGTCCTCCCGAATG	STAT1	AAGCCAAGTCACGTAGAATCGC<br>GCATCGTAGAGCCCGTCAGA
SMAD3	ACCACCAGATGAACCACAGCA<br>GGAGATGGAGCACCAGAAGG	HSP90B1	GATTGGGCAGTTTGGTGTCG<br>CCAGATATGCTGGGTATCGTTGT
GSK3B	AGGTCGTGTTAGGGGTCTTGC<br>CTTCTGGGTATTGGTTCACTTCA	RAC1	GACTCCCATCACCTACCCACA<br>GGAACCCAACATTTACAACAGC
AKT1	CCGTTAGGATTTCGTGTTTAGTTC<br>CCTATGTGAGGCTAATCGTTCTG	MAP2K2	AGGTCAGCATTGCGGTTCTC<br>GGATGTTGGATGGTTTCACG
